# The complete mitochondrial genome of the national bird of Peru: *Rupicola peruvianus* (Aves, Passeriformes, Cotingidae)

**DOI:** 10.1080/23802359.2019.1688721

**Published:** 2019-11-13

**Authors:** Danilo E. Bustamante, Jeffery R. Hughey, Jani E. Mendoza, Daniel Tineo, Jhordy Perez, Manuel Oliva, Santos Leiva, Martha S. Calderon

**Affiliations:** a Instituto de Investigación para el Desarrollo Sustentable de Ceja de Selva (INDES-CES), Universidad Nacional Toribio Rodríguez de Mendoza, Chachapoyas, Peru;; b Division of Mathematics, Science, and Engineering, Hartnell College, Salinas, CA, USA

**Keywords:** Amazonas, Andean Cock-of-the-Rock, mitogenome, phylogeny, Rupicola

## Abstract

*Rupicola peruvianus* Latham, known as the Andean Cock-of-the-Rock or locally as Tunqui, is distributed in the Andean cloud forests of South America from Venezuela to Bolivia. Here, we contribute to the bioinformatics and evolutionary systematics of the Cotingidae by performing high-throughput sequencing analysis on *R. peruvianus* from Luya, Amazonas, Peru. The *R. peruvianus* mitogenome is 17,035 base pairs (bp) in length and contains 37 genes (GenBank accession No. MN602289). The mitogenome is similar in structure and content to published mitogenomes from the neognathid orders Passeriformes and Falconiformes. Phylogenomic analysis of the *R. peruvianus* mitogenome situates it in a clade with the Pipridae, sister to the Tyrannidae. We anticipate that further mitogenome sequencing of the parvorder Tyrannida will improve the phylogenetic resolution and our understanding of the evolutionary history of this taxon.

*Rupicola peruvianus* is a vulnerable species distributed in the Andean cloud forests in South America from Venezuela to Bolivia (Ohlson et al. 2007). This essentially frugivorous species exhibits a striking sexual dimorphism (Show 1982). The male is characterized by a fan-shaped crest and brilliant orange plumage, while the female displays browner colors (Mahecha et al. 2018). Males spend much of their time displaying at leks where females select the one with which they will mate (Show 1982; Mahecha et al. 2018). After mating, females build the nest and rear the young (Show 1982). To contribute to the evolutionary systematics of the Cotingidae, and to advance the understanding of the taxonomy of *R. peruvianus*, this study characterized the complete mitochondrial genome of a male specimen of *R. peruvianus* from Luya, Amazonas, Peru (6°0′4″S, 78°7′42″W).

DNA was extracted from the feathers of the Andean Cock-of-the-Rock (Specimen Voucher: UFV-ZOO215) using the Quick-DNA Plant/Seed kit (Zymo Research, Irvine, CA, USA) following the manufacturer’s instructions. The 150 bp PE Illumina library construction and sequencing was performed by myGenomics, LLC (Alpharetta, GA, USA). The genome was assembled using default de novo settings in CLC Genomics Workbench 12.0 (QIAGEN Bioinformatics, Redwood City, CA, USA) and Sanger sequencing to close the gap in the control region using primers 15,455F 5′-TCCTAAACTTGCGCTCCGTT-3′ and 15,769R 5′-GTCCACAGCCTAAGACCCAC-3′ following the protocol of Bustamante et al. (2017). The mitogenome was confirmed using default mapping settings in Geneious Prime (Biomatters Ltd., Auckland, New Zealand). The genes were annotated with MITOS (Bernt et al. 2013) and manually using ORFfinder. The *R. peruvianus* mitogenome was aligned to other mitogenomes using MAFFT (Katoh and Standley 2013). The phylogenetic analysis was executed with RAxML-NG (Kozlov et al. 2019) with the GTR + gamma model and 1000 bootstraps. The tree was visualized with TreeDyn 198.3 at Phylogeny.fr (Dereeper et al. 2008).

The mitogenome of *R. peruvianus* is 17,035 bp in length and contains 37 genes. It has a slight A + T skewed (56.2%) and includes 22 tRNA (tRNA-Leu and tRNA-Ser occur in duplicate), 2 rRNA (rnl, rns), 13 genes involved in electron transport and oxidative phosphorylation, and 2 control regions (CRI, CRII). The mitogenome of *R. peruvianus* is similar in length, content, and organization to 24 Passeriformes and 10 Falconiformes characterized by Mackiewicz et al. (2019) as type GO-IV, which contains an abbreviated CRII.

This phylogenomic analysis represents the first for a species from the Contingidae. The analysis positions *R. peruvianus* in a clade with the Pipridae, sister in position to the Tyrannidae (Figure 1). A similar evolutionary relationship for these families was reported by Ericson et al. (2006) and Ohlson et al. (2013) based on nuclear and mitochondrial data. Further complete mitogenome sequencing of species classified to Cotingidae subfamilies and also to related families will help improve our understanding of the phylogenetics and taxonomy of Tyrannida.

**Figure 1.**
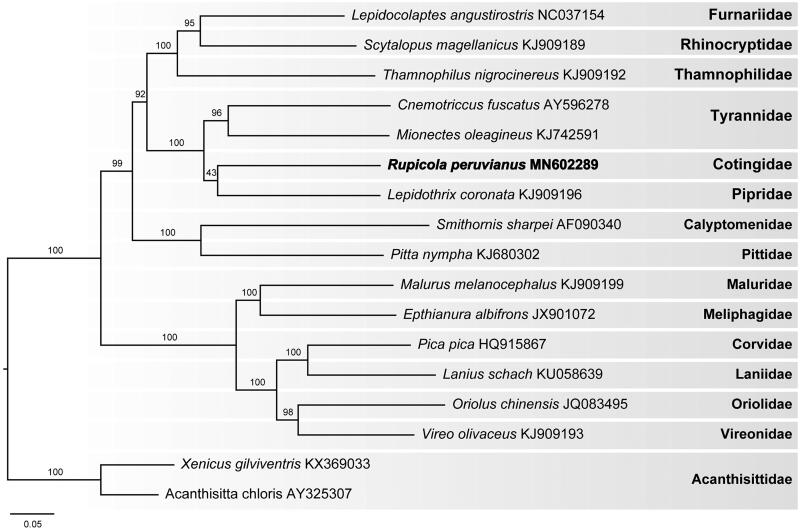
Maximum likelihood phylogram of *R. peruvianus* (MN602289) and related Passeriformes mitogenomes. Numbers along branches are RaxML bootstrap supports based on 1000 nreps. The legend below represents the scale for nucleotide substitutions.
